# Role of Melatonin in Apple Fruit during Growth and Ripening: Possible Interaction with Ethylene

**DOI:** 10.3390/plants11050688

**Published:** 2022-03-02

**Authors:** Antía Verde, Jesús M. Míguez, Mercedes Gallardo

**Affiliations:** 1 Departamento de Biología Vegetal, C.C. del Suelo, Universidade de Vigo, 36310 Vigo, Spain; averde@uvigo.es; 2 Departamento de Biología Funcional, C.C. de la Salud, Universidade de Vigo, 36310 Vigo, Spain; jmmiguez@uvigo.es

**Keywords:** apple, melatonin, fruit growth, ripening, ethylene

## Abstract

The role of melatonin during the growth and ripening of apple fruit was studied using local varieties. The evolution of the growth and ripening parameters, including fruit size and weight, firmness, color change, sugar content, and ethylene production, was different in the five varieties studied, with yellow apples (Reineta and Golden) initiating the ripening process earlier than reddish ones (Teórica, Sanroqueña, and Caguleira). Changes in the melatonin and melatonin isomer 2 contents during growth and ripening were studied in Golden apples, as was the effect of the melatonin treatment (500 µM, day 124 post-anthesis) on the apple tree. Melatonin content varied greatly, with higher value in the skin than in the flesh. In the skin, melatonin increased at day 132 post-anthesis, when ethylene synthesis started. In the flesh, melatonin levels were high at the beginning of the growth phase and at the end of ripening. Melatonin isomer 2 was also higher once the ripening started and when ethylene began to increase. The melatonin treatment significantly advanced the ethylene production and increased the fruit size, weight, sugar content, and firmness. The data suggest that melatonin stimulates fruit ripening through the induction of ethylene synthesis, while melatonin treatments before ripening improve the final fruit quality.

## 1. Introduction

Apples are one of the most widely grown and consumed fruits across the world. Currently, their production reaches 87 million tons, which makes them of great commercial importance. In addition to their nutritional value, many studies have highlighted the health-promoting properties of apples due to the presence of different active components, such as vitamins, minerals, and high concentrations of phenolic compounds [1,2,3]. Phenolic derivatives have strong antioxidant activity, so the consumption of foods rich in these compounds, including apples and apple products (apple juice, apple puree, and dried apple slices), has been associated with a reduced risk of cardiovascular and neurodegenerative diseases, diabetes, obesity, asthma, and cancer [3,4,5,6,7]. The high economic and nutritional value of apples, as well as their health-promoting properties, has prompted much research to focus on the physiological and molecular basis of apple development and ripening.

The growth and development of the apple fruit (*Malus domestica*, Borkh 1803) extends over approximately 150 days, depending on the variety. After pollination of the flower, seed development begins, which undergoes an active growth phase until the fruit reaches maximum size [7]. From a physiological point of view, apple fruit development may be divided into four partially overlapping stages: cell division, cell expansion, maturation, and ripening [8,9,10]. Each stage is under the control of multiple factors, both endogenous and exogenous. Among the former, hormones play a key role throughout the process of fruit development and ripening [7,10]. Thus, during the early stages of developmental, hormonal control over the cell cycle and energy supply are the determining factors affecting the final fruit size and quality [11]. The cell expansion phase is characterized by a strong uptake of water, together with the transport of sugars and other solutes into the vacuole, leading to a large increase in fruit volume and an accumulation of starch [9,10,12]. Finally, regarding the maturation stage, the apple acquires its final size and the competence to ripen, attaining this stage even without the need of the mother plant.

Fruit ripening is a complex process involving numerous metabolic, physiological, and structural changes that are tightly regulated at both the genetic and hormonal levels. In terms of hormones, ethylene plays a major role in controlling many of the processes leading to fruit ripening [10,13,14]. In so-called climacteric fruits, increased ethylene biosynthesis, together with an increased respiratory rate, characterize the onset of fruit ripening. The apple is a typical climacteric fruit. It ripens in approximately four weeks, with ethylene playing a very important regulatory role in determining the changes in flavor, aroma, color, and softening that lead to fruit ripening, thus determining its final quality [10,15,16]. Therefore, much work has focused on describing the changes in ethylene levels during ripening in various apple varieties [17]. In particular, a significant increase in ethylene production during early ripening has been described in some commercially widespread varieties such as Golden, Gala, and Fuji [16,18,19].

Melatonin (N-acetyl-5-methoxytryptamine) is an indoleamine synthesized from tryptophan metabolism, via serotonin, which is well known for its excellent antioxidant properties and high free radical-scavenging capacity. Melatonin was first discovered in the pineal gland of mammals, with important roles in the regulation of circadian rhythms, metabolism, and the immune system [20,21]. Subsequent studies have shown that melatonin is also present in a wide variety of plants and in all plant organs (seeds, leaves, stems, roots, flowers, and fruits), in which its levels vary widely [22,23,24,25]. Furthermore, melatonin has been suggested to be involved in many physiological functions in plants and to affect the biosynthetic pathways of other plant hormones, participating in important processes such as seed germination, root development, flowering, photosynthesis, senescence [26,27,28], and fruit ripening [29]. It also plays an important role in protecting cells against the oxidative damage caused by abiotic stresses [26,27,30,31].

Several studies have indicated that melatonin content varies with fruit development, increasing during the growth phases and generally decreasing during fruit ripening. This pattern of evolution of melatonin levels has been observed in grapes [32], tomatoes [33], and blackberries [34], although in cherries, the melatonin levels remain constant until the end of ripening [35]. Furthermore, the exogenous application of melatonin in grapevines [36], pears [37], and blackberries [38] has been shown to increase the fruit number, size, and weight, as well as the content of phenolic compounds. Regarding the possible regulatory role of melatonin in fruit ripening, the results obtained are contradictory. Thus, while in tomatoes and peaches exogenous melatonin application stimulates the ethylene levels and ripening-related parameters [39,40], in bananas, it delays senescence during the post-harvest period [41], which also occurs in some varieties of pears [31,37] and strawberries [42]. In cherries, the application of melatonin to the tree during the pre-harvest period delays ripening [35].

The presence of melatonin in apples has been known for years [43,44], having been described both in the fruit itself and in its juice, although the highest concentrations have been found in the skin [6]. However, there is very little information on the role of melatonin during apple growth and development, or on its relationship with ripening. Therefore, the aim of the present study was first to investigate changes in the melatonin levels in apples during growth and ripening in both the skin and flesh. In addition, by means of exogenous treatments, we investigated whether melatonin is involved in the ripening process of apples, as well as its possible interaction with ethylene and the processes that determine the final quality of the fruit.

## 2. Results

### 2.1. Experiment 1: Evolution of the Growth and Ripening Parameters, and Changes in the Melatonin Content

#### 2.1.1. Analysis of the Physiological Parameters of Apples

**Figure 1:**
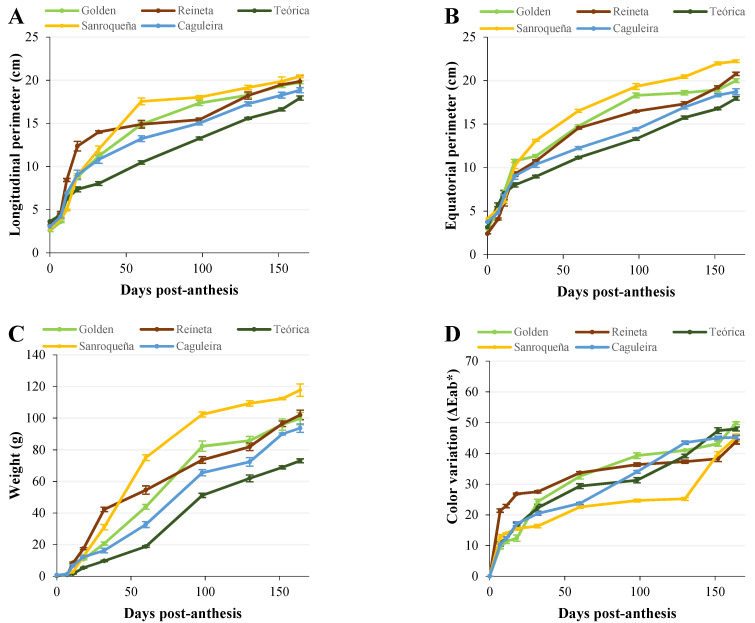
Evolution of the growth parameters in five apple varieties during the post-anthesis period. Longitudinal perimeter (**A**), equatorial perimeter (**B**), weight (**C**), and color variation (**D**). Error bars represent the standard error of the mean. Statistical significance is described in Table S1.

**Figure 2:**
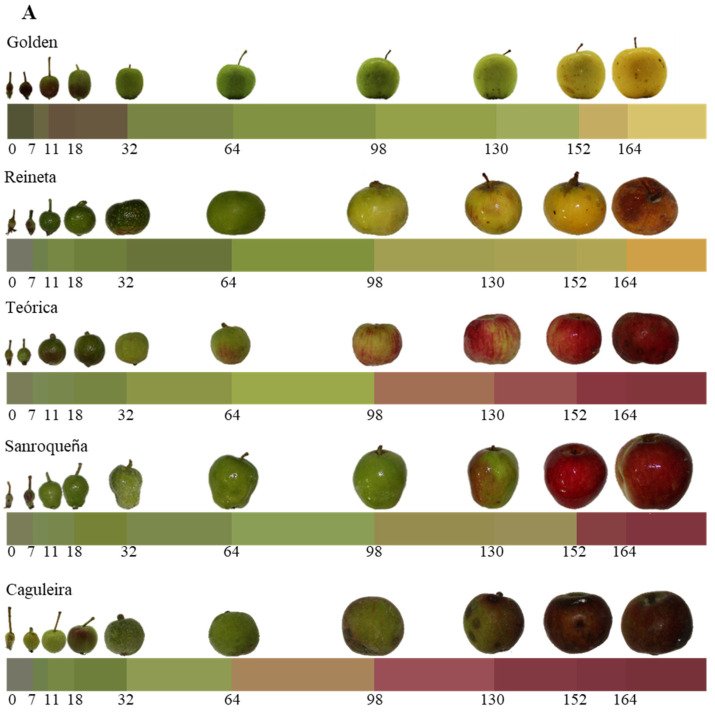

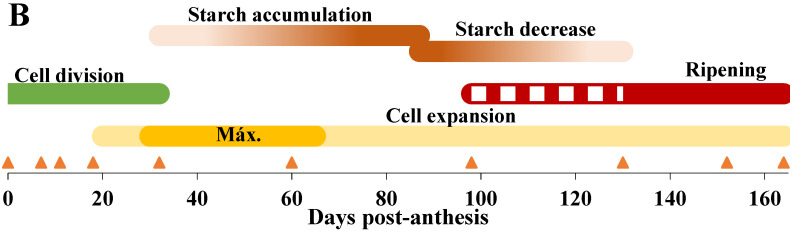
Evolution of the fruit growth and ripening of different apple varieties during the post-anthesis period indicated on the abscissa axis. (**A**) Pictures taken at each of the sampling stages, together with a representation of the variation of fruit color during growth and ripening. (**B**) Schematic diagram showing the main processes that occur during fruit development (adapted from Janssen et al. [9]). The orange triangles represent the sampling days throughout the post-anthesis period.

Figure 1 shows the evolution of the different growth and ripening parameters in the five apple varieties studied. Ten stages were established for this monitoring, from day 0 post-anthesis (fertilized flower) to day 164 (time when full pre-senescence ripening is reached). The morphological and size variations, such as the longitudinal (Figure 1A) and equatorial (Figure 1B) perimeters, as well as the weight variation (Figure 1C), showed a sigmoidal growth curve, which is typical of most fruits. Thus, their values increased significantly and exponentially in all of the apple varieties studied during almost the entire growth period, and this increase being very marked in the first stages (days 0–32 post-anthesis) that correspond to the phase of cell division and a high rate of cell expansion, as schematized in Figure 2. Likewise, in all of the varieties studied, the growth rate started to decline in the last stages (days 130, 152, and 164 post-anthesis), corresponding to the beginning of fruit ripening (Figure 2A).

In addition to the growth parameters, Figure 1 shows the variation in color over the period studied. The color underwent a natural change throughout the growth and development of the fruit in all apple varieties, which resulted in a greater difference in color the longer the time elapsed with respect to the initial measurement (t_0_). As shown in Figure 1D, those varieties in which ripening culminated in a golden-yellow coloring, such as Golden and Reineta (Figure 2A), showed a similar evolution with a gradual increase throughout fruit development. However, those apples where the final coloring after ripening reached reddish colors, such as Caguleira and Sanroqueña, showed much more pronounced patterns in color variation. Thus, in the Caguleira variety, a strong increase in color variation (ΔEab*) was observed throughout the whole period studied, starting with a reddish shade from day 64 post-anthesis and acquiring shades close to garnet or dark red at the end of ripening. The color difference for the Sanroqueña variety was much less pronounced, as this apple maintained a mostly greenish coloring during fruit growth and until the beginning of ripening (day 130 post-anthesis; Figure 2A), when a strong increase in the color difference was observed.

#### 2.1.2. Evolution of Ethylene Production

**Figure 3:**
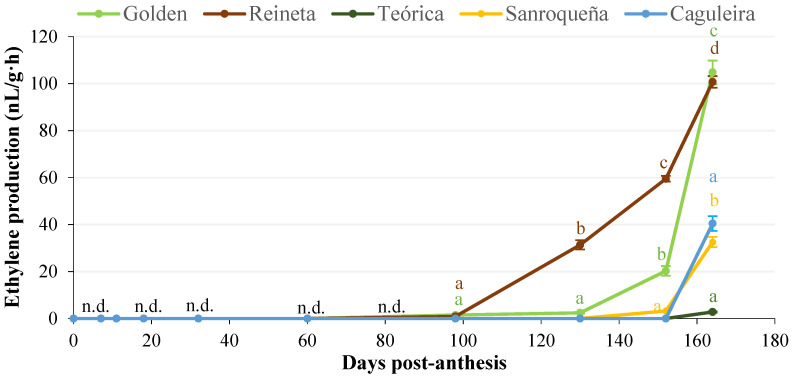
Evolution of ethylene production during apple fruit growth and ripening for the five varieties under study. Error bars represent the standard error of the mean. Different letters indicate significant differences between sampling days post-anthesis within each variety (*p* < 0.001). n.d., not detected.

Figure 3 shows the variation in ethylene production throughout the growth and ripening process of the fruit for the five apple varieties studied. The ethylene levels (nL/g·h) were undetectable during the initial phases of fruit development, and in the case of Golden and Reineta, were not measurable until 98 days post-anthesis, when the production of this phytohormone began. As expected for climacteric fruits, with the onset of apple fruit ripening, there was a drastic increase in the ethylene levels until the so-called climacteric peak was reached. In our study, the Reineta and Golden varieties showed a strong positive slope in ethylene production, which was more pronounced in Reineta, a variety with an earlier ripening. In neither of these two apple varieties was the climacteric maximum reached in the 164 days post-anthesis covered by our study. For the rest of the varieties under analysis, ethylene production was not detected until days 152 and 164 post-anthesis, so a longer follow-up time would have been necessary.

#### 2.1.3. Evolution of Melatonin and Melatonin Isomer 2 Contents

**Figure 4:**
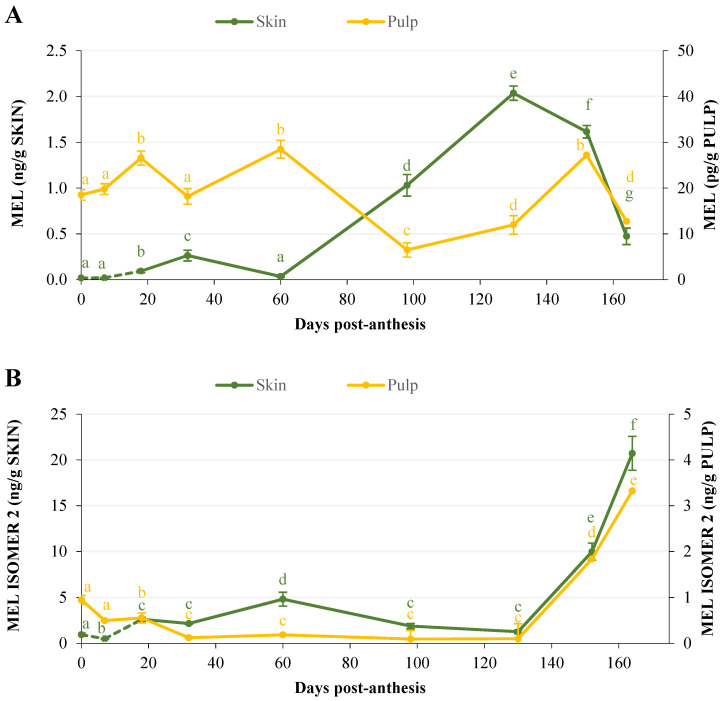
Content of melatonin (**A**) and its isomer 2 (**B**) in the skin and pulp throughout the growth and ripening in Golden apple fruit. Error bars represent the standard error of the mean. Different letters indicate significant differences between the post-anthesis sampling days within the same tissue (*p* < 0.001).

Figure 4 shows the variation in melatonin and melatonin isomer 2 contents in Golden apple skin and flesh during the growth and ripening processes. Melatonin content in the skin was approximately 60 times higher than in the flesh during the whole study period (Figure 4A), reaching 2 ng per gram of fresh tissue in the skin, compared to 30 pg per gram in the flesh. In the skin, the melatonin levels were low during the early stages of fruit growth (0–60 days post-anthesis), increasing sharply (more than 50-fold) until day 130 post-anthesis and then declining steadily until the end of the study. On the contrary, the melatonin levels quantified in the pulp did not show a defined pattern, ranging between 6 and 28 pg per gram of tissue during the whole period studied.

Figure 4B shows the changes in the melatonin isomer 2 levels in apple. Similar to melatonin, the levels of the isomer were higher (up to 6 times higher) in the skin than in the flesh, although in both tissues, they were several times higher than those of melatonin. The evolution of the melatonin isomer 2 content was very similar in the skin and pulp throughout the study period, with low levels up to day 130 post-anthesis and a significant increase thereafter until the end of the study, reaching 20.71 ng/g for skin and 3.32 ng/g for pulp.

### 2.2. Experiment 2: Effect of the In Vivo Application of Melatonin to the Tree upon Fruit Ripening

**Figure 5:**
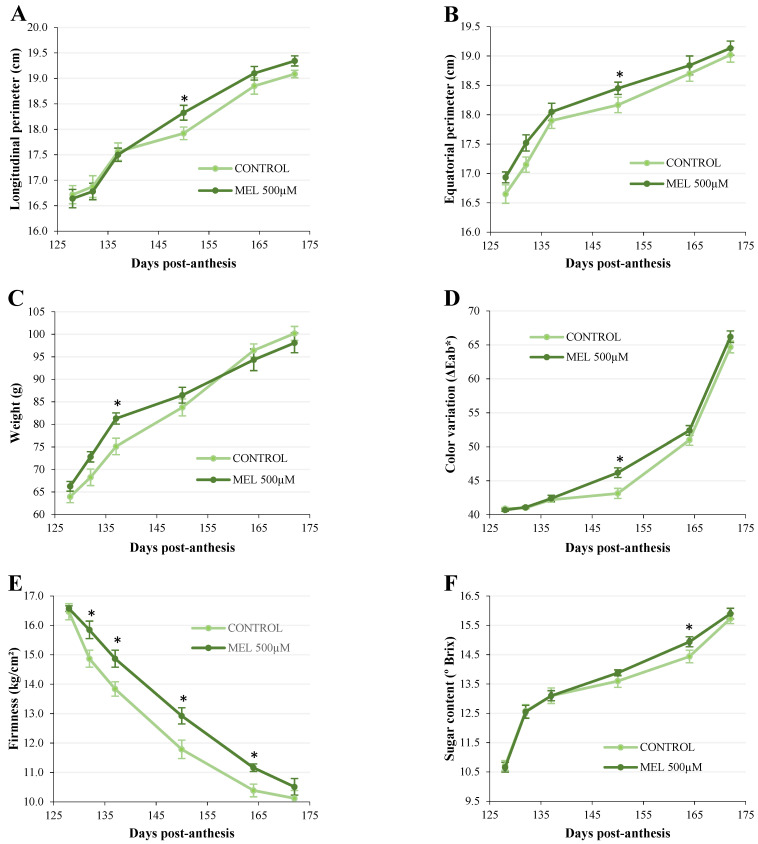
Effect of the in vivo application of melatonin (MEL 500 µM) on the growth parameters of a local Golden apple. Longitudinal perimeter (**A**), equatorial perimeter (**B**), weight (**C**), color variation (**D**), firmness (**E**), and sugar content (**F**) over 44 days, corresponding to the fruit ripening phase of the tree. Error bars represent the standard error of the mean. Asterisks indicate significant differences between treatments within the same time period (*p* < 0.05).

Figure 5 depicts the evolution of the different morphological and physiological parameters in Golden apple after in vivo application of melatonin (500 µM) to the apple tree, subsequently sampling six stages of fruit ripening. The longitudinal and equatorial perimeters (Figure 5A,B) in the fruit of apple trees treated with melatonin showed a slight increase in a good part of the period studied, being significant for both parameters on day 150 post-anthesis, with respect to the control group. This increase is in accordance with the variation in apple weight during this period (Figure 5C). Thus, melatonin (500 µM) caused an increase in weight from the beginning of the treatment, which was significant on day 137 post-anthesis (8.37% higher than the control), with no differences from this time until the end of the period studied.

The effect of melatonin on the physiological parameters most directly related to apple ripening is shown in Figure 5D–F. The color variation after melatonin application (500 µM) did not show significant differences, with the exception of day 150 post-anthesis, where the color change was more pronounced in the treated group compared to the control (Figure 5D). On the contrary, the presence of melatonin significantly delayed fruit softening, determined as the loss of fruit firmness in the ripening stages studied (Figure 5E). Finally, the sugar content (expressed as °Brix) increased throughout the ripening process, being slightly higher from day 137 post-anthesis in apples treated with melatonin (significant on day 164 post-anthesis) compared to the control group (Figure 5F).

**Figure 6:**
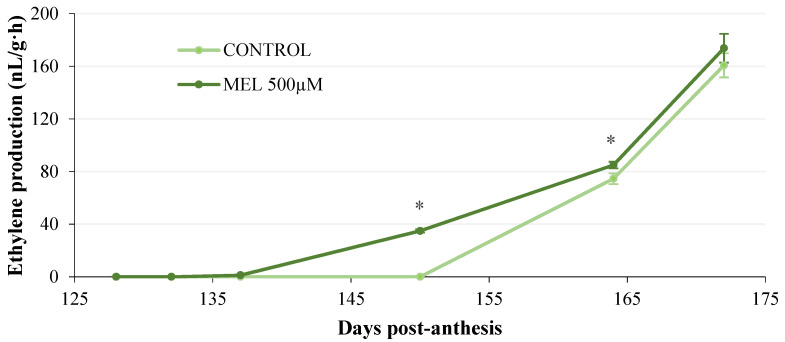
Effect of the in vivo application of melatonin (MEL 500 µM) on ethylene production during fruit ripening on the tree of a local Golden apple. Asterisks represent significant differences between treatments within the same time period (*p* < 0.05).

The ethylene production in Golden apples is shown in Figure 6. As indicated above, this variety showed typical climacteric behavior, with a continuous increase in the synthesis of the phytohormone as ripening progressed, reaching values close to 160 nL/g·h in the final stage (172 days post-anthesis). Melatonin treatment resulted in a 13-day advance in the onset of ethylene synthesis compared to untreated apples. Moreover, ethylene production was significantly higher in melatonin-treated apples up to day 165 post-anthesis. However, no significant differences were noticed in the final part of the study period compared to the control group.

## 3. Discussion

Apples are a fruit highly appreciated by consumers due to its healthy properties, hence its cultivation is widespread all over the world. There is a growing scientific interest in this fruit, both in terms of cultivation and post-harvest handling, as well as in improving the quality of the final product. The growth and development of fleshy fruits, such as apples, involves several stages that can be summarized as follows: (i) fruit setting; (ii) a phase of active growth by cell division and elongation when it reaches its final size; (iii) a phase in which the fruit acquires the competence to initiate the last stage of development; and (iv) ripening [7]. This last phase involves very important metabolic and physiological changes that result in the conversion of the green (inedible) fruit into an attractive fruit in terms of firmness, flavor, aroma, and color.

In the present study, the evolution of the morphological parameters that define fruit growth, together with the changes in coloring that take place during the ripening process, was investigated in five apple varieties obtained from local producers and grown under organic production labeling. Monitoring lasted from the fertilized flower stage (day 0 post-anthesis) until the fruit reached full ripening, prior to senescence (day 164 post-anthesis). All of the apple varieties studied showed similar behavior in terms of morphological and size variations over the period studied. The evolution of the longitudinal and equatorial perimeters that define the final shape of the apple, together with the weight, showed a typical sigmoidal growth curve that characterizes most fleshy fruits [45]. Accordingly, the initial growth rate increased exponentially during the first stages (days 0–32 post-anthesis), a period in which the cell division phase and the peak of maximum cell expansion take place, as described by Janssen et al. [9] in Royal Gala apples. In addition, as expected, with the onset of ripening (from approximately day 130 post-anthesis, depending on the variety), there was a slowdown in growth.

Fruit ripening is an irreversible, highly coordinated, and genetically programmed process involving a series of physiological, biochemical, and organoleptic changes, leading to variations in color, firmness, flavor, aroma, and nutritional composition [10,46,47,48]. In our study, changes in the coloring of the different apple varieties tested were evident throughout the process of fruit growth and ripening. These changes are produced by the degradation of chlorophylls and the synthesis of new pigments, among which, in apples, anthocyanins and carotenoids predominate [7,10]. However, this process varies strongly depending on the apple variety, whose shades can evolve toward golden-yellow tones that respond to a greater accumulation of carotenoids, as observed here in the Golden and Reineta varieties. If the synthesis of anthocyanins predominates, the apples take on reddish or even maroon tones, as has been found in the Teórica, Sanroqueña, and Caguleira varieties.

Ethylene is considered the main hormone regulating ripening. In climacteric fruits such as apples, ethylene coordinates the biochemical and developmental pathways that affect changes in color, texture, nutritional quality, and aroma of the ripening fruit. These fruits are characterized by a sharp increase in ethylene levels until they reach what is known as the climacteric peak [7,39,49]. In our study, the yellow-colored varieties (Reineta and Golden) initiated the ripening process earlier, which correlated with an increase in ethylene synthesis, with Reineta showing the most intense and incipient increase. Although our study extended up to 164 days post-analysis, we could not determine the climacteric maximum for any of the apple varieties analyzed, so a longer time would be necessary. Nevertheless, the observed changes are in general agreement with the available literature on the variations that occur in ethylene levels during ripening in apples [17]. In this regard, a significant increase in ethylene production has been described in the Golden, Gala, and Fuji varieties, which, as in our study, coincide with the onset of ripening [16,18,19]. Likewise, our results agree with those of the evolution of ripening in apples of the Gala variety [19], in which the climacteric maximum of ethylene synthesis could not be concluded either.

Melatonin is a multifunctional molecule that is widely present in plants. It has also been described in a wide variety of fruits such as tomatoes, grapes, cherries, bananas, and pears [24,26]. However, there are few references to the presence and levels of melatonin in apples [43,44], which, in any case, concern only the ripe fruit. The present work describes, for the first time, the evolution of melatonin content and one of its isomers during the growth and ripening process of the Golden apple variety, which has been used as a study model. Both skin and flesh tissue were screened for the presence of melatonin and melatonin isomer 2 in all the growth and maturation stages studied. It should be noted that the melatonin content was significantly higher in the skin (2 ng/g) than in the flesh (30 pg/g fresh weight), as was melatonin isomer 2, which showed tenfold higher levels, reaching 22 ng/g in the skin and 3.3 ng/g in the flesh. A study by Zhang et al. [6] on 18 apple varieties, not including the Golden variety, also described a higher melatonin content in the skin compared to the flesh, albeit with higher melatonin levels than observed herein. A higher melatonin content in the skin than in the flesh was also found by Vitalini et al. [50] in grapes.

Our study shows strong changes in melatonin levels during apple growth. In the skin, the melatonin content exhibited a strong increase at the end of the cell expansion phase, reaching a maximum on day 132 post-anthesis. This is the time when fruit ripening and color changes begin, which, in turn, coincides with the start of ethylene synthesis for this apple variety. However, the melatonin content in the flesh fluctuated more, reaching the highest levels in the initial stages of growth and at the end of the ripening process. As for melatonin isomer 2, both in the skin and in the flesh, its synthesis was maximal at the end of the period studied, once ripening had begun and coinciding with the start of ethylene synthesis.

These data suggest that there is active melatonin synthesis at different stages of fruit growth and ripening, which may be regulated by the factors involved in the control of these processes. At present, the role of melatonin during the process of fruit growth and ripening is not known, although it has been suggested that the content of this indolamine varies substantially [51]. Thus, in agreement with our results, the melatonin content in grapes increased during fruit growth, reaching a maximum when the color change started, decreasing thereafter with fruit ripening [32]. Likewise, Vitalini et al. [50] reported that the melatonin levels in grape skin were highest at the time of fruit color change. A similar pattern was also obtained for tomatoes, with the highest melatonin concentrations detected when the fruit turned red [33]. In our study, this pattern was observed for Golden apples, where the maximum melatonin levels in the skin were found when the color changed from green to yellowish (day 132 post-anthesis).

In a similar work to ours on melatonin evolution during cherry fruit development, Zhao et al. [52] observed that the melatonin concentration increased considerably during the elongation and cell expansion phase, suggesting that melatonin might be involved in these processes. Similarly, Xia et al. [53], also in cherries, indicated that the melatonin levels decreased during fruit ripening, also supporting that the melatonin concentration is highly regulated by plant developmental processes. Taking all of this into account, it can be inferred that the melatonin concentration in plant cells is highly regulated by plant developmental processes. Furthermore, our results suggest that melatonin plays different roles in the skin and in the flesh, with changes in its levels evolving differently. In the pulp, the melatonin content was highest during the rapid growth phase, when cell division and expansion predominate, in agreement with that reported in cherries by Zhao et al. [52]. In the skin, however, the highest melatonin levels coincided with the onset of ripening (color change) and the increase in ethylene content, suggesting that melatonin may play a role in stimulating the synthesis of the phytohormone. This agrees with that proposed by Sun et al. [39,54] in tomatoes, although in their study, no distinction was made between the skin and the pulp of the fruit. In contrast, other authors have observed that the exogenous application of melatonin in bananas [41] or pears [31] reduces ethylene production and delays senescence during post-harvest. In our study in apples, it seems that increased melatonin levels may modulate fruit ripening by acting specifically on the induction of ethylene production from fruit skin cells. Furthermore, the high levels of melatonin isomer 2 detected in both the apple skin and flesh, coinciding with the onset of ethylene synthesis, suggest that not only melatonin but other isomeric forms of this molecule reported in plants [55,56] may have some role in the regulation of ripening through their interaction with ethylene synthesis. However, it is still too early to conclude anything solid, and further studies on the role of melatonin and its isomeric forms in apples are needed.

To deepen the understanding of the possible role of melatonin in apple ripening and its relationship with ethylene production, in a second experiment, a melatonin treatment was applied to Golden apple trees with fruit in the green stage (124 post-anthesis), just before the onset of ripening. Ethylene production and the parameters related to fruit quality (variations in size and weight, color, firmness, and sugar content) were quantified over six ripening stages by sampling apples from the plant itself. The results showed that the application of melatonin to the apple tree led to a 13-day advance in ethylene production compared to the control treatment, while the levels of this phytohormone were also higher. Melatonin also increased the fruit weight during the first 12 days after its application on the tree, and this increase was significant on day 137 post-anthesis (8.37% vs. control). A slight increase in fruit size was also observed after melatonin treatment, which was significant on day 150 post-anthesis. These results are consistent with those found in other studies following the application of exogenous melatonin to growing fruit. Thus, treatment of the tree with melatonin increased the fruit weight by 6.6% in grapes [57] and by 47.8% in pears [37]. Increases in fruit number, weight, and size were also obtained in blackberries after melatonin application [38]. In addition, Okatan et al. [58] reported that after melatonin spraying in apples, the soluble solid content (°Brix), as well as phenols and organic acids, increased. In our case, the sugar content, determined as °Brix, was also slightly higher after melatonin application, being significant 150 days post-anthesis, in association with a more rapid change in apple coloring. It should also be borne in mind that we applied the treatment to the apple tree branches when the fruit was very advanced in its growth (124 post-anthesis) and with a single dose of melatonin, so the effect may have been less evident than if it had been administered at the beginning of the fruit’s growth or with successive doses. The most evident effect of melatonin was on fruit firmness, causing an increase throughout the ripening period studied, compared to the control group, which would lead to a delay in apple senescence. Similar results to these were reported in tomatoes [39], peaches [40], and grapes [32] after the application of exogenous melatonin to the fruit, with increases in ethylene synthesis and the negative regulatory effects on fruit senescence. In our case, it appears that melatonin application to apple branches promoted fruit ripening through the induction of ethylene synthesis, accelerating the apple color change and sugar content, while delaying senescence. All of these effects contributed to the improvement of the final quality of the fruit, resulting in apples with a higher degree of firmness and a greater weight and size, which is a clear benefit for the producer and the consumer. However, more detailed studies on the role of melatonin in apple ripening are needed, focusing, in particular, on a possible interaction with ethylene synthesis, as well as on the cell signaling and gene expression mechanisms that mediate the physiological response to this phytohormone.

## 4. Materials and Methods

### 4.1. Plant Material and Experimental Design

#### 4.1.1. Plant Material

Five different varieties of local apples were harvested from the village of A Estrada (Spain). Specifically, the varieties Reineta, Teórica, Sanroqueña, and Caguleira were from the certified organic apple orchard Torres de Moreda (Callobre, A Estrada), while the Golden apples were harvested from other farmers in the same area. The identification of the apple trees from each variety was established on the basis of documentation provided by the growers. Apples were harvested between May and November 2019.

#### 4.1.2. Experiment 1: Evolution of the Apple Fruit Growth and Ripening Parameters, and Changes in Melatonin Content

The process of fruit development and ripening was characterized for the different apple varieties. Ten samplings were scheduled, the first one corresponding to the stage of fertilized flower (day 0 post-anthesis) and the following ones to different moments of growth and ripening of the fruit, specifically, days 7, 11, 18, 32, 60, 98, 130, 152, and 164 post-anthesis. Depending on the harvesting stage, between 10 and 15 samples of each variety were collected, taking those fruits with the greatest morphological homogeneity (size, weight, coloring, etc.).

Several morphological parameters were measured at each stage, including longitudinal and equatorial perimeters, and weight and color variation, as well as the determination of ethylene production. The apples were frozen at –80 °C until subsequent analysis of the melatonin content, which was carried out only in the Golden apples.

#### 4.1.3. Experiment 2: Effect of the In Vivo Application of Melatonin to the Tree on Fruit Ripening

Golden apples were used as a model for this experiment. When they reached physiological maturity (approximately day 124 post-anthesis), two groups of six trees each (control and treatment) were selected, with the same solar orientation. The apple trees were then sprayed once at a distance of approximately 25 cm with a melatonin solution (500 µM) or with distilled water in the case of the control group. After the treatments, six samplings were carried out during the ripening process, harvesting on days 128, 132, 137, 150, and 164 until day 172 post-anthesis, immediately before the onset of senescence.

Each sampling day, the longitudinal and equatorial perimeters, weight, firmness, color variation, sugar content, and ethylene production were measured. Afterward, the apples were frozen at –80 °C for further analysis.

### 4.2. Determination of the Apple Development and Ripening Parameters

Measurement of the perimeters, weight, firmness, sugar content, and color variation

The longitudinal and equatorial perimeters and weight variation of the apples were measured as usual. Firmness was assessed using a penetrometer (Texture Analyzer, DT120), equipped with a 6 mm-diameter plunger. Two measurements were performed on each apple, one in a random area of its largest diameter and the other on the opposite side. The results are expressed in kilograms per square centimeter (kg/cm^2^).

The sugar content was determined by a refractometer (Atago, ATC-1; McCormick Fruit Tech, Fukaya-shi, Saitama, Japan) and the results are expressed in °Brix.

The color variation was evaluated by determining the CIE color space parameters L* a* and b* [59], where L* is the lightness coordinate, a* is the red (+a*)-green (−a*) coordinate, and b* is the yellow (+b*)-blue (−b*) coordinate. The skin color was determined using a colorimeter (PCE-SM2), taking two measurements on opposite sides of the fruit, in the equatorial region. The results are expressed as the color variation according to the following equation:

ΔEab* = [(L*t_0_ − L*t_n_)^2^ + (a*t_0_ − a*t_n_)^2^ + (b*t_0_ − b*t_n_)^2^]^1/2^

where t_0_ is the day of initial measurement and t_n_ is each of the measurement days.

### 4.3. Analysis of Ethylene Production

Individual apple samples (at least six replicates) were placed in a hermetically sealed container equipped with septa, and then incubated for 1 h in the darkness at 25 °C. After incubation, 1 mL was withdrawn from the internal atmosphere of the container and injected into a gas chromatographer (Hewlett-Packard 6890 Plus, Agilent, Santa Clara, CA, USA), equipped with a flame ionization detector with electronically controlled pneumatics and a capillary injector for operations with and without splitting (0–100 psi), following a method modified from de Dios et al. [60]. The ethylene levels released by each apple were expressed in nanoliters of ethylene per gram of apple per hour (nL·g^−1^·h^−1^).

### 4.4. Determination of Melatonin and Its Isomer Content

#### 4.4.1. Sample Extraction

Melatonin content was determined in Golden apples, which were used as a model to study its evolution during the development and ripening of the fruit. Melatonin was quantified in both the skin and flesh at the developmental stages described above. The apple skin was carefully obtained with a scalpel on an ice tray, avoiding any contamination of the pulp. One gram of this tissue was placed in liquid nitrogen and subsequently homogenized in a porcelain mortar until a powder was obtained. Methanol (3 mL) was added and the sample was homogenized vigorously. In parallel, for the flesh analysis, triangular segments of the fruit were cut in order to obtain a homogeneous sample from each apple. Then, 2.5 g of flesh were homogenized and methanol (7.5 mL) was added and mixed vigorously. The samples were then placed in an ultrasonic bath (Branson M 3510) for 30 min and subsequently shaken in a programmable rotator (Multi Bio RS-24) for 30 min at 4 °C in dark conditions. The tubes were centrifuged for 25 min at 3000× *g* and the supernatants were dried in a vacuum concentrator (Speed-Vac; V-Al program at 30 °C). Finally, the samples were resuspended in 0.5 mL of 5% acetonitrile acidified with formic acid (pH 2.5).

The extraction of melatonin was performed using organic solvents, as previously described by Verde et al. [61]. Thus, 2 mL of chloroform was added to each sample and vortexed vigorously for 2 min. After centrifugation at 3000× *g* and 4 °C for 10 min, the aqueous phase was removed by aspiration. Then, 0.5 mL of NaOH (0.2 N) was added to the chloroformic phase and vortexed for 1 min. After centrifugation for 5 min, the obtained aqueous phase was removed and the volume of the chloroformic phase of each sample was quantified. The samples were dried under vacuum and resuspended in 100 µL of a 5% acetonitrile solution with formic acid 0.1% (pH 2.5).

#### 4.4.2. Analysis of Melatonin and Its Isomer by HPLC

Quantification of melatonin and melatonin isomer 2 (N-acetyl-6-methoxytryptamine) was performed using the reversed-phase HPLC technique with fluorescence detection, as previously reported [61] but with some modifications. The chromatography system consisted of a compact HP 1100 device equipped with a quaternary pump (HP 1311A), a degasser (HP G1322A), and a fluorescence detector (HP G131A) set at 285/345 excitation/emission wavelengths. Chromatographic separation was carried out on a Supercosil LC-18-DB column (15 cm × 4.6 mm, 5 μm) held at 25 °C in a column oven (Jasco CO-4060). The mobile phase was obtained using a tertiary gradient consisting of (A) 0.1% of formic acid, (B) 60% of acetonitrile containing 0.1% (*v*:*v*) formic acid, and (C) 90% of acetonitrile containing 0.1% (*v*:*v*) formic acid. The elution sequence was: 10% B–0% C (0 min), 45% B–0% C (12 min), 100% C (14–15 min), and 10% B–0% C (17 min) to recover the initial conditions. All analyses were performed at a flow rate of 1.0 mL/min. The acquisition and integration of chromatograms was carried out with HP 1100 ChemStation software. Identification and quantification of melatonin and melatonin isomer 2 was carried out by comparison of the retention times of the peaks with those of the reference substances and by standard addition to samples under several chromatographic conditions that affected the retention times. Under the routine analysis conditions, the retention times for melatonin and melatonin isomer 2 were 9.5 min and 10.1 min, respectively. The results are expressed as nanograms (ng) or picograms (pg) of melatonin or melatonin isomer per gram of tissue.

### 4.5. Statistical Analysis

The results are expressed as the mean ± standard error of the mean (SEM) of the data for each of the parameters analyzed in the experimental groups. In those cases in which the conditions of equality of variance or normality failed, a logarithmic transformation of the data was performed previously. One-way ANOVA was used for between-groups comparisons, with the main variable ‘days post-anthesis’ (Experiment 1) or ‘treatment’ (Experiment 2). When necessary, the analysis was completed with a Student–Newman–Keuls test. The significance level was set at *p* < 0.001 (Experiment 1) or *p* < 0.05 (Experiment 2).

## 5. Conclusions

The presence of melatonin in fruits has been widely studied, but there are few relevant reports on its possible role in fruit development and ripening. In this work, the evolution of the growth and ripening parameters, including ethylene synthesis, during the post-anthesis period was studied in several local apple varieties, showing that yellow apples (Reineta and Golden) initiate the ripening process earlier than red apples (Teórica, Caguleira, San Roqueña). In Golden apples, the melatonin levels were detected very early, on day 0 post-anthesis, varying throughout the growth and ripening of the fruit but always being higher in the skin than in the flesh. Melatonin levels increased sharply at the beginning of the fruit ripening phase, coinciding with the increase in ethylene synthesis and with the change in apple coloration. Interestingly, melatonin isomer 2 in the both skin and flesh also showed higher values during the ripening phase. These results clearly point to a physiological role of melatonin in fruit ripening, so an interaction with ethylene in this phase is possible. In fact, treatment of Golden apple trees with melatonin at a pre-ripening stage brought forward the onset of the ethylene peak and increased its levels, thus advancing fruit ripening. This effect could mediate the changes promoted by melatonin in apples, which involved (i) an increase in size and weight; (ii) an increase in sugar content; (iii) a faster color change; and (iv) a significant increase in fruit firmness during the whole period after melatonin treatment. Taken together, these results allow us to conclude that melatonin leads to an acceleration of the apple fruit ripening parameters, as well as a possible delay of the senescence process.

## Data Availability

All data generated or analysed during this study are included in this published article and its Supplementary Information Files.

## Supplementary Materials

The following supporting information can be downloaded at https://www.mdpi.com/article/10.3390/plants11050688/s1, Table S1: Evolution of the growth parameters (longitudinal and equatorial perimeters, weight and color variation) in five apple varieties during the post-anthesis period.
