# Supplementary material for: Role of Melatonin in Apple Fruit during Growth and Ripening: Possible Interaction with Ethylene
Source: Plants (Basel). 2022 Mar 2;11(5):688. doi: 10.3390/plants11050688 (PMC8912437; doi:10.3390/plants11050688)
Supplement: Supplementary file 1 [file plants-11-00688-s001.zip › plants-1590392-supplementary.pdf]

Table S1. Evolution of the growth parameters (longitudinal and equatorial perimeters, weight and color variation) in five apple varieties during the post-anthesis period. Data are expressed as mean ± SEM (n = 5). Different letters indicate significant differences between sampling days post-anthesis within each variety, $p < 0.001$.

	Days Post-Anthesis									
	**0**	**7**	**11**	**18**	**32**	**60**	**98**	**130**	**152**	**164**
	**Longitudinal Perimeter (cm)**									
Golden	2,59 ± 0,13 ^{a}	3,57 ± 0,07 ^{b}	6,30 ± 0,15 ^{c}	8,76 ± 0,19 ^{d}	11,23 ± 0,28 ^{e}	14,89 ± 0,28 ^{f}	17,36 ± 0,30 ^{g}	18,27 ± 0,25 ^{h}	19,38 ± 0,22 ^{i}	19,76 ± 0,17 ^{i}
Reineta	3,23 ± 0,15 ^{a}	4,65 ± 0,14 ^{b}	8,44 ± 0,17 ^{c}	12,36 ± 0,56 ^{d}	14,00 ± 0,14 ^{e}	14,90 ± 0,44 ^{e}	15,43 ± 0,20 ^{f}	18,23 ± 0,41 ^{g}	19,51 ± 0,13 ^{h}	19,85 ± 0,69 ^{h}
Teórica	3,63 ± 0,13 ^{a}	4,42 ± 0,33 ^{b}	6,30 ± 0,19 ^{c}	7,36 ± 0,29 ^{d}	8,04 ± 0,22 ^{e}	10,47 ± 0,20 ^{f}	13,25 ± 0,17 ^{g}	15,58 ± 0,11 ^{h}	16,62 ± 0,17 ^{i}	17,93 ± 0,24 ^{j}
Sanroqueña	2,77 ± 0,03 ^{a}	3,93 ± 0,09 ^{b}	4,97 ± 0,09 ^{c}	8,97 ± 0,22 ^{d}	12,00 ± 0,37 ^{e}	17,55 ± 0,39 ^{f}	18,03 ± 0,20 ^{f}	19,15 ± 0,26 ^{g}	19,88 ± 0,50 ^{g}	20,45 ± 0,17 ^{g}
Caguleira	3,05 ± 0,03 ^{a}	4,18 ± 0,19 ^{b}	6,90 ± 0,12 ^{c}	9,03 ± 0,54 ^{d}	10,83 ± 0,45 ^{e}	13,23 ± 0,33 ^{f}	15,03 ± 0,21 ^{g}	17,26 ± 0,23 ^{h}	18,28 ± 0,34 ^{i}	18,85 ± 0,30 ^{i}
	**Equatorial Perimeter (cm)**									
Golden	3,00 ± 0,12 ^{a}	5,06 ± 0,11 ^{b}	7,20 ± 0,21 ^{c}	10,77 ± 0,17 ^{d}	11,33 ± 0,17 ^{e}	14,76 ± 0,16 ^{f}	18,31 ± 0,28 ^{g}	18,60 ± 0,24 ^{g}	18,97 ± 0,27 ^{g}	19,99 ± 0,22 ^{h}
Reineta	2,40 ± 0,00 ^{a}	4,08 ± 0,12 ^{b}	5,94 ± 0,36 ^{c}	9,26 ± 0,26 ^{d}	10,75 ± 0,14 ^{e}	14,55 ± 0,12 ^{f}	16,48 ± 0,09 ^{g}	17,33 ± 0,25 ^{h}	19,23 ± 0,18 ^{i}	20,78 ± 0,19 ^{j}
Teórica	3,15 ± 0,17 ^{a}	5,74 ± 0,22 ^{b}	7,05 ± 0,31 ^{c}	8,00 ± 0,24 ^{d}	8,98 ± 0,14 ^{e}	11,15 ± 0,11 ^{f}	13,30 ± 0,16 ^{g}	15,75 ± 0,18 ^{h}	16,78 ± 0,13 ^{i}	17,96 ± 0,20 ^{j}
Sanroqueña	4,13 ± 0,15 ^{a}	5,17 ± 0,18 ^{b}	6,03 ± 0,15 ^{c}	10,23 ± 0,35 ^{d}	13,13 ± 0,11 ^{e}	16,53 ± 0,17 ^{f}	19,33 ± 0,34 ^{g}	20,45 ± 0,19 ^{h}	21,98 ± 0,18 ^{i}	22,23 ± 0,15 ^{i}
Caguleira	3,75 ± 0,10 ^{a}	4,83 ± 0,09 ^{b}	6,73 ± 0,20 ^{c}	9,03 ± 0,38 ^{d}	10,38 ± 0,36 ^{e}	12,25 ± 0,17 ^{f}	14,40 ± 0,16 ^{g}	16,95 ± 0,21 ^{h}	18,33 ± 0,18 ^{i}	18,73 ± 0,34 ^{i}
	**Weight (g)**									
Golden	0,34 ± 0,02 ^{a}	0,78 ± 0,05 ^{b}	8,61 ± 0,65 ^{c}	10,72 ± 0,38 ^{c}	20,67 ± 0,98 ^{d}	43,97 ± 1,50 ^{e}	82,25 ± 3,24 ^{f}	85,71 ± 2,64 ^{f}	95,90 ± 3,54 ^{fg}	99,72 ± 3,96 ^{g}
Reineta	0,25 ± 0,03 ^{a}	0,92 ± 0,06 ^{b}	8,43 ± 0,40 ^{c}	17,47 ± 0,62 ^{d}	42,28 ± 1,41 ^{e}	54,54 ± 2,65 ^{f}	73,60 ± 2,10 ^{g}	81,90 ± 2,36 ^{g}	96,39 ± 1,76 ^{h}	101,97 ± 3,01 ^{h}
Teórica	0,38 ± 0,02 ^{a}	0,80 ± 0,03 ^{b}	1,58 ± 0,15 ^{c}	5,35 ± 0,28 ^{de}	9,69 ± 0,45 ^{e}	18,78 ± 0,57 ^{f}	51,17 ± 1,32 ^{g}	61,98 ± 2,15 ^{h}	68,93 ± 0,98 ^{hi}	73,08 ± 1,24 ^{i}
Sanroqueña	0,62 ± 0,03 ^{a}	1,50 ± 0,04 ^{b}	2,78 ± 0,23 ^{b}	12,68 ± 1,07 ^{c}	31,07 ± 1,66 ^{d}	75,11 ± 1,81 ^{e}	102,30 ± 1,55 ^{f}	109,30 ± 1,66 ^{fg}	112,35 ± 0,68 ^{fg}	117,69 ± 3,91 ^{g}
Caguleira	0,67 ± 0,02 ^{a}	1,27 ± 0,10 ^{b}	6,74 ± 0,42 ^{c}	12,24 ± 0,51 ^{d}	16,27 ± 1,47 ^{e}	32,70 ± 1,87 ^{f}	65,51 ± 1,96 ^{g}	72,40 ± 2,75 ^{g}	89,98 ± 0,62 ^{h}	93,63 ± 2,69 ^{h}
	**Color Variation (ΔEab*)**									
Golden	-	9,49 ± 0,59 ^{b}	11,32 ± 0,63 ^{c}	12,41 ± 1,00 ^{c}	24,33 ± 0,78 ^{d}	32,57 ± 0,90 ^{e}	39,28 ± 0,92 ^{f}	41,02 ± 0,17 ^{f}	43,14 ± 0,75 ^{f}	49,80 ± 0,46 ^{g}
Reineta	-	21,40 ± 0,57 ^{b}	22,84 ± 0,57 ^{c}	26,83 ± 0,25 ^{d}	27,51 ± 0,39 ^{d}	33,70 ± 0,39 ^{e}	36,37 ± 0,60 ^{f}	37,32 ± 0,52 ^{f}	38,28 ± 0,93 ^{f}	43,82 ± 0,76 ^{g}
Teórica	-	10,90 ± 0,43 ^{b}	12,25 ± 0,58 ^{c}	16,42 ± 0,91 ^{d}	22,30 ± 1,09 ^{e}	29,37 ± 0,83 ^{f}	31,28 ± 0,84 ^{f}	39,17 ± 0,58 ^{g}	47,36 ± 0,95 ^{h}	47,91 ± 0,66 ^{h}
Sanroqueña	-	12,88 ± 0,76 ^{b}	13,98 ± 0,15 ^{b}	15,52 ± 0,51 ^{c}	16,33 ± 0,54 ^{c}	22,55 ± 0,47 ^{d}	24,66 ± 0,46 ^{e}	25,22 ± 0,49 ^{e}	39,97 ± 0,64 ^{f}	45,11 ± 1,20 ^{g}
Caguleira	-	10,29 ± 0,55 ^{b}	12,25 ± 0,58 ^{c}	17,09 ± 0,61 ^{d}	20,45 ± 0,56 ^{e}	23,74 ± 0,32 ^{f}	34,01 ± 0,41 ^{g}	43,47 ± 0,51 ^{h}	44,99 ± 0,58 ^{h}	45,15 ± 0,66 ^{h}
